# Supplementary material for: How well are non-communicable disease services being integrated into primary health care in Africa: A review of progress against World Health Organization’s African regional targets
Source: PLoS One. 2020 Oct 22;15(10):e0240984. doi: 10.1371/journal.pone.0240984 (PMC7580905; doi:10.1371/journal.pone.0240984)
Supplement: Attachment — Submitted filename: Response to Reviewers_PHC integration_OCt 2nd, 2020.docx [file pone.0240984.s002.docx]

Date: October 2, 2020

**Author Response Letter: PLOS ONE [PONE-D-20-19186R1 and PONE-D-20-19186R2]**

Dear Dr Pastakia,

Thank you for reviewing the revised manuscript on “How well are non-communicable disease services being integrated into primary health care in Africa: a review of progress against World Health Organization’s African regional targets”. We have made the revision based on the points the reviewer raised and uploaded a clean and tracked change manuscript.

**Reviewer' comments**

1. *Please provide the full name of WHO PEN (**WHO Package of Essential Noncommunicable disease interventions), in addition to the abbreviation, the first time you mentioned it in the manuscript(introduction).*

- We have now included the full name of WHO PEN in the revised manuscript. Please see page 3, line 22.

2. *Table 1 - Target 3 - URL for "Data was extracted from WHO essential medicine and health product information portal" - The page cannot be found. Please double check your URL.*

- The correct URL has included now, where it shows the National (all countries) medicines list/formulation/standard treatment guideline portal. Please see Table 1, Target 3.

3. *Table 3 - Indicator "Availability of essential medicine list at national and PHC level," please change your subtext to reflect essential medicine LIST. For example, "Essential medicine LIST available nationally and at the PHC level."*

- We have made the correction in the revised manuscript. Please see Table 3.

**Editor’s comment [PONE-D-20-19186R2]**

1. *Your ethics statement should only appear in the Methods section of your manuscript. If your ethics statement is written in any section besides the Methods, please move it to the Methods section and delete it from any other section. Please ensure that your ethics statement is included in your manuscript, as the ethics statement entered into the online submission form will not be published alongside your manuscript.*

- *We have moved the ethics statement to the method section of the manuscript. Please see page 11, line 24.*

Thank you again for your feedback and your time to review our paper. Please contact me if you have any further questions.

Best regards,

Azeb Gebresilassie Tesema

ORCID iD: https://orcid.org/0000-0003-0618-4499
